# A phase II study of irinotecan and pegylated liposomal doxorubicin in platinum-resistant recurrent ovarian cancer (Tohoku Gynecologic Cancer Unit 104 study)

**DOI:** 10.1007/s00280-017-3363-0

**Published:** 2017-06-27

**Authors:** Tadahiro Shoji, Eriko Takatori, Hideo Omi, Masahiro Kagabu, Tatsuya Honda, Masayuki Futagami, Yoshihito Yokoyama, Michiko Kaiho, Hideki Tokunaga, Takeo Otsuki, Tadao Takano, Nobuo Yaegashi, Takanobu Kojimahara, Tsuyoshi Ohta, Satoru Nagase, Shu Soeda, Takafumi Watanebe, Hiroshi Nishiyama, Toru Sugiyama

**Affiliations:** 1 Department of Obstetrics and Gynecology, Iwate Medical University School of Medicine, 19-1 Uchimaru, Morioka, 020-8505 Japan; 2 Department of Obstetrics and Gynecology, Morioka Japanese Red Cross Hospital, 6-1-1 Sanbonyanagi, Morioka, 020-8560 Japan; 3 Department of Obstetrics and Gynecology, Hirosaki University School of Medicine, 53 Honcho, Hirosaki, 036-8563 Japan; 4 Department of Obstetrics and Gynecology, Tohoku University Graduate School of Medicine, 1-1 Seiryouchou, Sendai, 980-8574 Japan; 5 Department of Obstetrics and Gynecology, Okitama Public General Hospital, 2000 Nishiotsuka, Kawanishimachi, Higashiokitamagun, 992-0601 Japan; 6 Department of Obstetrics and Gynecology, Faculty of Medicine, Yamagata University, 2-2-2 Iidanishi, Yamagata, 990-9585 Japan; 7 Department of Obstetrics and Gynecology, Fukushima Medical University School of Medicine, 1 Hikarigaoka, Fukushima, 960-1295 Japan; 8 Department of Obstetrics and Gynecology, Iwaki Kyouritsu Hospital, 16 Kusehara, Uchigomimayamachi, Iwaki, 973-8555 Japan

**Keywords:** Recurrent ovarian cancer, Chemotherapy, CPT-11, PLD

## Abstract

**Purpose:**

We report a phase II clinical study of the combination of irinotecan (CPT-11) and pegylated liposomal doxorubicin (PLD) in platinum- and taxane-resistant recurrent ovarian cancer, based on the recommended doses determined in a phase I trial.

**Methods:**

PLD was administered intravenously at a dose of 30 mg/m^2^ on day 3. CPT-11 was administered intravenously at a dose of 80 mg/m^2^ on days 1 and 15, according to the recommendations of the phase I study. A single course of chemotherapy lasted 28 days, and patients underwent at least 2 courses until disease progression. The primary endpoint was antitumor efficacy, and the secondary endpoints were adverse events, progression-free survival (PFS), and overall survival (OS).

**Results:**

The response rate was 32.3% and the disease control rate was 64.5%. Grade 3 and 4 neutropenia, anemia, and a decrease in platelet count were observed in 17 (54.9%), 3 (9.7%), and 1 patient (3.2%), respectively. In terms of grade 3 or higher non-hematologic toxicities, grade 3 nausea occurred in 1 patient (3.2%), vomiting in 3 patients (9.7%), and grade 3 diarrhea and fatigue in 1 patient (3.2%). The median PFS and OS rates were 2 months and not reached, respectively. Of the 11 patients with a treatment-free interval (TFI) of ≥3 months, the response rate was 63.3%, and the median PFS was 7 months.

**Conclusions:**

The treatment outcomes for the 31 patients enrolled in this study were unsatisfactory. However, sub-analysis suggested that patients with a TFI of ≥3 months had a good response rate and PFS. This suggests that CPT-11/PLD combination therapy may be a chemotherapy option for platinum-resistant recurrent ovarian cancer.

## Introduction

In the treatment of patients with platinum-resistant recurrent ovarian cancer, it is essential to select drugs that do not demonstrate cross-resistance to the initial therapy. In Western countries, the type I DNA topoisomerase inhibitors such as topotecan [1], pegylated liposomal doxorubicin (PLD) [2], and gemcitabine [3] are used as monotherapies. In a Japanese phase II study of patients with ovarian cancer, previously treated with chemotherapy that included platinum-based agents, PLD was reported to achieve an overall response rate of 21.9% (27.3% [3/11] in the platinum-sensitive group and 21.0% [13/62] in the platinum-resistant group) [2]. In a phase III non-inferiority study comparing PLD with topotecan, PLD achieved an overall response rate of 19.7%, a median progression-free survival (PFS) of 16.1 weeks, and a mean survival time (MST) of 60.0 weeks; in particular, in patients with platinum-resistant tumors, the response rate was 12.3%, the median PFS was 9.1 weeks, and the MST was 35.6 weeks [4]. These results suggested that PLD could be a promising therapeutic agent for recurrent ovarian cancer. On the other hand, irinotecan hydrochloride (CPT-11), an anticancer agent developed in Japan, acts by inhibiting topoisomerase I. In a study of platinum-resistant recurrent ovarian cancer treated with CPT-11 monotherapy (100 mg/m^2^), the response rate was 29%; the tumor growth inhibition rate (complete response [CR] + partial response [PR] + no change) was 61%; the median time to progression was 17 weeks; and the MST was 8 months, exhibiting favorable results [5]. Sugiyama et al. reported that CPT-11/cisplatin combination therapy was effective as a second-line chemotherapy for recurrent ovarian cancer after treatment with a platinum agent [5], which means that CPT-11 may be effective for platinum- and taxane-resistant tumors.

We previously conducted a phase I study of the combination of CPT-11 and PLD in patients with platinum- and taxane-resistant recurrent ovarian cancer and reported that the recommended doses of CPT-11 and PLD were 80 and 30 mg/m^2^, respectively [6]. Here, we report a phase II study based on the recommended doses determined in the phase I study.

## Subjects and methods

### Study population

Approval was obtained from the intramural ethics committee of each study center. A multi-center clinical study was conducted in patients with recurrent ovarian cancer who were enrolled in the study between April 2013 and March 2015 and who met the following criteria: (1) ovarian cancer confirmed by histological or cytological diagnosis, (2) recurrence <6 months after previous chemotherapy, (3) a measurable or evaluable lesion (including CA 125 level), (4) European Oncology Cooperative Group performance status 0–2, (5) age between 20 and 75 years, (6) expected survival time of >2 months, (7) no effect on major organ functions (white blood cell count ≥3000/mm^3^, neutrophil count ≥1500/mm^3^, platelet count ≥10,0000/mm^3^, total bilirubin ≤1.5 mg/dL), and (8) informed consent provided. Exclusion criteria were: (1) serious complication(s), (2) evident pulmonary fibrosis or interstitial pneumonitis, (3) pleural or cardiac effusion necessitating prompt local treatment, (4) brain metastasis requiring prompt treatment, (5) diarrhea (watery stool), (6) intestinal paralysis or intestinal obstruction, (7) active infection requiring treatment with antimicrobial agents, and (8) patients considered inappropriate as subjects by the physician in charge for any other reason.

### Protocol

PLD was administered intravenously at a dose of 30 mg/m^2^ on day 3. CPT-11 was administered intravenously at a dose of 80 mg/m^2^ on days 1 and 15, according to the recommendation of the phase I study. A single course of chemotherapy lasted 28 days and, as a general rule, patients underwent at least two courses, until progressive disease (PD).

#### Sample size

In previous studies of patients with platinum-resistant recurrent ovarian cancer, the response rates associated with multiple-agent chemotherapy with CPT-11 ranged from 29 to 50% [7, 8, 9]. On the other hand, PLD monotherapy was reported to have response rates between 8.3 and 21.9% [2, 10, 11]. If the CPT-11/PLD combination therapy resulted in a response rate well below that of the above therapies, it would be assumed that the combination therapy had no clinical significance. Therefore, we set the expected response rate and the threshold response rate of the CPT-11/PLD combination therapy in this study at 40 and 20%, respectively. The required sample size was calculated as 36 using a double-sided *α* error of 0.05 and *β* error of 0.2. The planned sample size was estimated at 40, including patients who might become ineligible.

#### Criteria for changing the dosing schedule

If any of the following conditions applied, CPT-11 administration on day 15 was postponed: (1) white blood cell count ≤2000/mm^3^, (2) neutrophil count ≤1000/mm^3^, (3) platelet count ≤75,000/mm^3^, or (4) grade 1 or higher diarrhea. Once recovery was confirmed, the drug was administered on day 22. If recovery from the condition was not seen on day 22, the second CPT-11 administration was skipped (not to be administered on day 29). The criteria for proceeding to the second and subsequent courses were: (1) white blood cell count ≥3000/mm^3^, (2) neutrophil count ≥1500/mm^3^, (3) platelet count ≥100,000/mm^3^, (4) total bilirubin ≤1.5 mg/dL, (5) diarrhea grade 0, and (6) grade 1 or lower hand–foot syndrome and mucositis. If the patient met any of the above criteria, treatment was postponed for up to 14 days for recovery. If, after 14 days, recovery from the above conditions was not observed (4 weeks for hand-and-foot syndrome or stomatitis), the treatment was discontinued. If the severity of hand–foot syndrome or mucositis remained at grade 2 or higher after a 14-day postponement, PLD on day 3 in the next treatment course was skipped.

#### Criteria for dose reduction

The doses of CPT-11 and PLD in the next course were reduced according to the severity of any adverse reactions that occurred in the previous course. If grade 4 leukopenia, grade 4 neutropenia, or grade 3 thrombocytopenia was observed in the preceding course, the CPT-11 dosage was reduced by 10 mg/m^2^ and PLD was reduced by 7.5 mg/m^2^. If grade 2 or higher diarrhea, spasmodic abdominal pain, or watery stool was observed, the CPT-11 dose was reduced by 10 mg/m^2^. If grade 3 hand–foot syndrome or mucositis was observed, the PLD dose was reduced by 7.5 mg/m^2^, regardless of whether or not these conditions improved before the start of the next course.

### Endpoints/variables

The primary endpoint was antitumor efficacy, and the secondary endpoints were adverse events, PFS, and overall survival (OS). The antitumor effect was evaluated via imaging after every two courses. For evaluation of the antitumor effect, the best response rate was calculated according to the RECIST (Response Evaluation Criteria in Solid Tumors) version 1.0 guidelines. Adverse events were evaluated according to the National Cancer Institute Common Toxicity Criteria version 4.0.

### Statistical analysis

PFS and OS were calculated from the chemotherapy start date to the documented date of progression, death, or last follow-up, whichever occurred first. Impact on survival was assessed by constructing Kaplan–Meier curves with a log-rank test. The Chi-square test and the log-rank test were used to compare and analyze patient characteristics between the phase I and II groups, and between patients with a <3- and ≥3-month treatment-free interval (TFI). All reported significance values were two tailed at a level of 0.05.

## Results

### Patient background characteristics

Table 1 shows the background characteristics of the 31 patients enrolled in this study from April 2013 through March 2015. All patients had been treated with taxane- or platinum-based agents as part of their previous therapy. Twenty patients (64.5%) had a TFI <3 months.

**Table 1:** Patient characteristics (*N* = 31)

Age (year)	
Median	56
Range	38–74
PS	
0	25
1	6
FIGO stage	
I	3
II	0
III	20
IV	8
Histological type	
Serous	20
Mucinous	1
Clear cell	8
Unknown	2
Previous regimens	
1	14
2	10
3≤	7
Last regimen	
Paclitaxel/carboplatin	23
Docetaxel/carboplatin	3
Docetaxel/gemcitabine	2
Gemcitabine	2
Paclitaxel	1
TFI (months)	
<3	20
3≤, 6<	11

*TFI* treatment-free interval

### Antitumor effect

Table 2 shows the antitumor effects of the treatment. Partial response (PR), stable disease (SD), and progressive disease (PD) were observed in 10 (32.3%), 10 (32.3%), and 11 (35.5%) patients, respectively. The response rate was 32.3% and the disease control rate was 64.5%. However, no patient achieved complete response (CR).

**Table 2:** Response

	*N*	%
CR	0	0
PR	10	32.3
SD	10	32.3
PD	11	35.5
Overall response	10	32.3
Disease control	20	64.5

*CR* complete response, *PR* partial response, *SD* stable disease, *PD* progressive disease

### Adverse events

Grades 3 and 4 neutropenia were observed in 11 (35.5%) and 6 (19.4%) patients, respectively. Grade 3 anemia was observed in 3 patients (9.7%). A grade 3 decrease in platelet count was observed in 1 patient (3.2%). In terms of grade 3 or higher non-hematologic toxicities, grade 3 nausea occurred in 1 patient (3.2%), vomiting in 3 patients (9.7%), and grade 3 diarrhea and fatigue in 1 (3.2%) patient (Table 3).

**Table 3:** Toxicity

	Grade 1	Grade 2	Grade 3	Grade 4	Grade 3≤ (%)
Hematologic toxicity					
Leucopenia	2	14	8	2	10 (32.3)
Neutropenia	0	7	11	6	17 (54.8)
Anemia	13	11	3	0	3 (9.7)
Thrombocytopenia	6	1	1	0	1 (3.2)
Non-hematologic toxicity					
Nausea	16	3	1	0	1 (3.2)
Vomiting	6	1	3	0	3 (9.7)
Diarrhea	1	2	1	0	1 (3.2)
Hand–foot	3	3	0	0	0 (0)
Mucositis	12	1	0	0	0 (0)
Appetite loss	10	7	0	0	0 (0)
Fatigue	8	3	1	0	1 (3.2)

### Administration status

In total, 119 treatment courses were administered to the 31 patients. Over the course of the study, 19 treatment cycles were postponed (16.0%). The reasons for postponement were as follows: neutrophil count <1500/mm^3^ (15 cycles); grade 2 hand–foot syndrome (1 cycle); and the discretion of the attending physician (3 cycles).

CPT-11 on day 15 was postponed in 3 cycles (2.5%). The reasons for postponement were neutrophil count <1000/mm^3^ (2 cycles) and the discretion of the attending physician (1 cycle). CPT-11 on day 15 was skipped in 5 cycles (4.2%). The reasons included lack of neutrophil count recovery (1 cycle), lack of platelet count recovery (1 cycle), and the discretion of the attending physician (3 cycles). CPT-11 and PLD doses were reduced in 1 patient owing to grade 4 neutropenia in the previous course. Grade 4 neutropenia was also observed in 5 other patients; all 5 patients experienced PD and their regimens were changed.

### PFS and OS

**Fig. 1:**
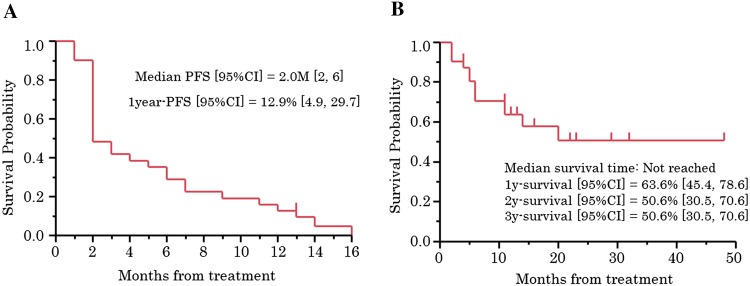
Survival. **a** Progression-free survival. **b** Overall survival

Median PFS was 2 months (95% confidence interval [CI] 2–6) and PFS at 1 year was 12.9% (95% CI 4.9–29.7) (Fig. 1a). The median OS was not reached, and the survival rate at 1 year was 63.6% (95% CI 45.4–78.6) (Fig. 1b).

## Discussion

We previously reported that the response rate and disease control rate of platinum-resistant recurrent ovarian cancer to CPT-11/VP16 combination therapy were 50.0 and 83.3%, respectively [9]. In a phase I study of 12 patients, the response rate and disease control rate were 58.3% (including 1 patient who achieved CR) and 75.0%, respectively [6]. Of the 31 patients in the present phase II study, the response rate and disease control rate were 32.2 and 64.5%, respectively, which did not meet our expectations. For further analysis, we compared the backgrounds between the 12 patients enrolled in the phase I study and the 31 patients enrolled in the present phase II study. Between the two patient groups, there were no differences in the proportion of PS 1, incidence of clear cell carcinoma, or the proportion of patients treated with ≥3 regimens. A significant difference was observed between the two groups regarding the proportion of patients with a TFI of <3 months (Table 4). We, therefore, compared the results between patients with a TFI <3 months and those with a TFI of 3–6 months in the present study. The comparisons between the 20 patients with a TFI of <3 months and the 11 patients with a TFI of 3–6 months showed that the response rates were 15.0 and 63.3%, respectively; the disease control rates were 55.0 and 81.8%, respectively; the median PFS rates were 2 months (95% CI 2–3) and 7 months (95% CI 2–13), respectively (*P* = 0.039, hazard ratio [HR] 0.48); and the 2-year survival rates were 36.4% (95% CI 16.1–63.0) and 80.8% (95% CI 47.2–95.2), respectively (*P* = 0.092, HR 0.29) (Table 5). We hypothesized that the large differences in the response, PFS, and OS rates between the phase I and phase II studies may be related to the differences in TFI between the two groups: in the phase I study, 4 patients (25.0%) had a TFI <3 months, while 20 patients (64.5%) in the phase II study had a TFI <3 months. Therefore, the proportion of patients with a TFI of <3 months was higher in the phase II study. Based on these results, we consider that CTP-11/PLD combination therapy can suppress tumor progression in patients with a TFI of 3–6 months, and may represent a second-line chemotherapy treatment option for platinum-resistant recurrent ovarian cancer, because it is associated with only mild adverse drug reactions. To confirm the treatment efficacy, further studies of its efficacy in platinum-resistant recurrent ovarian cancer with a TFI of 3–6 months, after excluding refractory cases, are necessary. The results of a questionnaire survey administered to member institutions of the Japanese Gynecologic Oncology Group (JGOG) in 2014 showed that PLD monotherapy was used as second-line therapy for platinum-resistant recurrent ovarian cancer in 47% of these institutions. Therefore, we advocate conducting a future phase III study, based on the results of this phase II study, on a nationwide basis in cooperation with societies such as the JGOG. It is also our view that the usefulness of CPT-11/PLD combination therapy should be verified by a superiority trial comparing PLD monotherapy as a control arm and CPT-11/PLD combination therapy as an experimental arm.

**Table 4:** Difference of the background in phase I and II study

	Phase I (*N* = 12)	Phase II (*N* = 31)	*P* value
ECOG performance status 1*	1	6	0.37
Clear cell carcinoma*	3	8	0.92
TFI <3 months*	4	20	0.04
Previous regimens 3≤*	5	7	0.21

*TFI* treatment-free interval

* Chi-square test

**Table 5:** Comparison of treatment results due to the difference in the TFI

TFK (months)	<3 (*N* = 20)	3<, <6 (*N* = 11)	*P* value
Response rate (%)*	15.0	63.3	0.0007
Disease control rate (%)*	55.0	81.8	0.016
Median PFS (95% CI) (months)**	2 [2, 3]	7 [2, 11]	0.039
2-year survival rate (95% CI) (%)**	36.4 (16.1, 63.0)	80.8 (47.2, 95.2)	0.092

*TFI* treatment-free interval, *PFS* progression-free survival

* Chi-square test, ** log-rank test

This 2-year study included only 31 patients, although the target sample size was 40. The small sample size was at least partly attributable to the difficulty in registering patients in some institutions. We presumed that approximately 5 patients with platinum-resistant recurrent ovarian cancer would attend each institution annually; however, no patients enrolled in 3 of the 7 institutions participating in the TGCU (Tohoku Gynecologic Cancer Unit) study group. This might have been due to the shortage of obstetricians and gynecologists in Japan, where many of them have to work very hard and they are very busy. We need to be more aware of the importance of clinical studies and greater efforts to establish scientific evidence are needed. Another limitation of this study was the cost of the clinical data management center. We realized that we needed to extend the registration period to reach the target sample size of 40 patients, but we decided to terminate the study owing to the cost of the extension. This is not a particular problem for our study group (TGCU). In Japan, clinical trials are being conducted in collaboration with clinical data management centers to ensure the quality of the studies. Since the cost of clinical data management centers is a huge expense, securing finance for projects is an important issue that needs to be resolved.

Recent studies have reported the use of molecular targeted drugs for treatment of platinum-resistant recurrent ovarian cancer. In the AURELIA (Avastin Use in Platinum-Resistant Epithelial Ovarian Cancer) trial, the median PFS of single-agent chemotherapy with bevacizumab was 6.7 months, and the HR was 0.48, compared with single-agent chemotherapy alone [12]. However, the molecular targeted drugs such as bevacizumab are expensive and pose a significant financial burden on patients. In Japan, conventional anticancer drugs have an advantage over molecular targeted drugs from a health economics perspective. For example, if a regimen used in the AURELIA trial, paclitaxel plus bevacizumab, PLD plus bevacizumab, or topotecan plus bevacizumab, was administered to a patient with a height of 160 cm, weight of 60 kg and body surface area of 1.6 m^2^, for 6 cycles, it would cost approximately 34,000 dollars (2,800,000 Japanese yen), 60,000 dollars (4,900,000 Japanese yen), or 43,000 dollars (3,600,000 Japanese yen), respectively. In contrast, CPT-11/PLD combination therapy (for 6 cycles) costs approximately 24,000 dollars (2,000,000 Japanese yen). We consider that CPT-11/PLD combination therapy can contribute to the reduction of health costs. Given that platinum-resistant recurrent ovarian cancer cannot be completely cured, chemotherapy has an important role in maintaining the quality of life and reducing the financial burden of patients. More recently, anti-PD-1 antibodies have become a treatment option in ovarian cancer, in addition to molecular targeted drugs such as olaparib [13], trebananib [14], and pazopanib [15]. Hamanishi et al. reported that the response rate and disease control rate of nivolumab in platinum-resistant recurrent ovarian cancer were 15 and 45%, respectively [16]. Following this pioneer study, a clinical trial of an anti-PD-1 antibody is currently being conducted in Japan. However, like molecular targeted drugs, anti-PD-1 antibodies are very expensive. CPT-11/PLD combination therapy has both cost and safety (less severe adverse drug reactions) advantages. We recommend that the usefulness of anticancer drugs should be reevaluated from economical and safety perspectives.

## References

[1] Aoki D, Katsumata N, Nakanishi T, Kigawa J, Fujiwara K, Takehara K, Kamiura S, Hiura M, Hatae M, Sugiyama T, Ochiai K, Noda K (2011). A phase II clinical trial of topotecan in Japanese patients with relapsed ovarian carcinoma. Jpn J Clin Oncol.

[2] Katsumata N, Fujiwara Y, Kamura T, Nakanishi T, Hatae M, Aoki D, Tanaka K, Tsuda H, Kamiura S, Takehara K, Sugiyama T, Kigawa J, Fujiwara K, Ochiai K, Ishida R, Inagaki M, Noda K (2008). Phase II clinical trial of pegylated liposomal doxorubicin (JNS002) in Japanese patients with mullerian carcinoma (epithelial ovarian carcinoma, primary carcinoma of fallopian tube, peritoneal carcinoma) having a therapeutic history of platinum-based chemotherapy: a phase II study of the Japanese Gynecologic Oncology Group. Jpn J Clin Oncol.

[3] Yoshino K, Hiramatsu K, Enomoto T, Fujita M, Ueda Y, Kimura T, Kobayashi E, Kiyohara Y, Tsutsui T, Kimura T (2012). Salvage chemotherapy using gemcitabine for taxane/platinum-resistant recurrent ovarian cancer: a single institutional experience. Anticancer Res.

[4] Gordon AN, Fleagle JT, Guthrie D, Parkin DE, Gore ME, Lacave AJ (2001). Recurrent epithelial ovarian carcinoma: a randomized phase III study of pegylated liposomal doxorubicin versus topotecan. J Clin Oncol.

[5] Matsumoto K, Katsumata N, Yamanaka Y, Yonemori K, Kohno T, Shimizu C, Andoh M, Fujiwara Y (2006). The safety and efficacy of the weekly dosing of irinotecan for platinum- and taxanes-resistant epithelial ovarian cancer. Gynecol Oncol.

[6] Sugiyama T, Yakushiji M, Nishida T, Ushijima K, Okura N, Kigawa J, Terakawa N (1998). Irinotecan (CPT-11) combined with cisplatin in patients with refractory or recurrent ovarian cancer. Cancer Lett.

[7] Shoji T, Takatori E, Kaido Y, Omi H, Yokoyama Y, Mizunuma H, Kaiho M, Otsuki T, Takano T, Yaegashi N, Nishiyama H, Fujimori K, Sugiyama T (2014). A phase I study of irinotecan and pegylated liposomal doxorubicin in recurrent ovarian cancer (Tohoku Gynecologic Cancer Unit 104 study). Cancer Chemother Pharmacol.

[8] Nishio S, Sugiyama T, Shouji T, Yoshizaki A, Kitagawa R, Ushijima K, Kamura T (2007). Pilot study evaluating the efficacy and toxicity of irinotecan plus oral etoposide for platinum- and taxane-resistant epithelial ovarian cancer. Gynecol Oncol.

[9] Shoji T, Takatori E, Omi H, Kumagai S, Yoshizaki A, Yokoyama Y, Mizunuma H, Fujimoto T, Takano T, Yaegashi N, Tase T, Nakahara K, Kurachi H, Nishiyama H, Sugiyama T (2011). Phase II clinical study of the combination chemotherapy regimen of irinotecan plus oral etoposide for the treatment of recurrent ovarian cancer (Tohoku Gynecologic Cancer Unit 101 Group Study). Int J Gynecol Cancer.

[10] Mutch DG, Orlando M, Goss T, Teneriello MG, Gordon AN, McMeekin SD, Wang Y, Scribner DR, Marciniack M, Naumann RW, Secord AA (2007). Randomized phase III trial of gemcitabine compared with pegylated liposomal doxorubicin in patients with platinum-resistant ovarian cancer. J Clin Oncol.

[11] Gordon AN, Granai CO, Rose PG, Hainsworth J, Lopez A, Weissman C, Rosales R, Sharpington T (2000). Phase II study of liposomal doxorubicin in platinum- and paclitaxel-refractory epithelial ovarian cancer. J Clin Oncol.

[12] Pujade-Lauraine E, Hilpert F, Weber B, Reuss A, Poveda A, Kristensen G, Sorio R, Vergote I, Witteveen P, Bamias A, Pereira D, Wimberger P, Oaknin A, Mirza MR, Follana P, Bollag D, Ray-Coquard I (2014). Bevacizumab combined with chemotherapy for platinum-resistant recurrent ovarian cancer: the AURELIA open-label randomized phase III trial. J Clin Oncol.

[13] Ledermann J, Harter P, Gourley C, Friedlander M, Vergote I, Rustin G, Scott CL, Meier W, Shapira-Frommer R, Safra T, Matei D, Fielding A, Spencer S, Dougherty B, Orr M, Hodgson D, Barrett JC, Matulonis U (2014). Olaparib maintenance therapy in patients with platinum-sensitive relapsed serous ovarian cancer: a preplanned retrospective analysis of outcomes by BRCA status in a randomised phase 2 trial. Lancet Oncol.

[14] Monk BJ, Poveda A, Vergote I, Raspagliesi F, Fujiwara K, Bae DS, Oaknin A, Ray-Coquard I, Provencher DM, Karlan BY, Lhommé C, Richardson G, Rincón DG, Coleman RL, Herzog TJ, Marth C, Brize A, Fabbro M, Redondo A, Bamias A, Tassoudji M, Navale L, Warner DJ, Oza AM (2014). Anti-angiopoietin therapy with trebananib for recurrent ovarian cancer (TRINOVA-1): a randomised, multicentre, double-blind, placebo-controlled phase 3 trial. Lancet Oncol.

[15] Kim JW, Mahner S, Wu LY, Shoji T, Kim BG, Zhu JQ, Takano T, Park SY, Kong BH, Wu Q, Wang KL, Ngan HY, Liu JH, Wei LH, Mitrica I, Zhang P, Crescenzo R, Wang Q, Cox CJ, Harter P, du Bois A (2015) Pazopanib maintenance therapy in East Asian Women with advanced epithelial ovarian cancer: results from AGO-OVAR16 and an East Asian Study. Int J Gynecol Cancer (**epub ahead of print**) 10.1097/IGC.0000000000000602 26588236

[16] Hamanishi J, Mandai M, Ikeda T, Minami M, Kawaguchi A, Murayama T, Kanai M, Mori Y, Matsumoto S, Chikuma S, Matsumura N, Abiko K, Baba T, Yamaguchi K, Ueda A, Hosoe Y, Morita S, Yokode M, Shimizu A, Honjo T, Konishi I (2015). Safety and antitumor activity of anti-PD-1 antibody, nivolumab, in patients with platinum-resistant ovarian cancer. J Clin Oncol.

